# Unraveling Charging and Discharging Processes in Organic Radical‐Based Electrodes: A Hierarchical Molecular and Quantum Mechanical Approach

**DOI:** 10.1002/cssc.202502645

**Published:** 2026-03-15

**Authors:** Clara Zens, Georgina E. Shillito, Christian Friebe, Stephan Kupfer

**Affiliations:** ^1^ Institute of Physical Chemistry Friedrich Schiller University Jena Jena Germany; ^2^ Helmholtz Institute for Polymers in Energy Applications Jena (HIPOLE Jena) Jena Germany; ^3^ Helmholtz‐Zentrum Berlin für Materialien und Energie Berlin Germany

**Keywords:** charge transfer, molecular dynamics, organic radical batteries, TEMPO

## Abstract

Organic batteries represent a promising class of energy storage materials, due to their mechanical flexibility and sustainability. Typically, stable radicals, lacking intrinsic conductivity, are utilized as redox‐active materials. A recently introduced strategy to overcome this shortcoming is to incorporate stable radicals, i.e., (2,2,6,6‐tetramethylpiperidin‐1‐yl)oxyl (TEMPO), into a polythiophene backbone. Thereby, an electrode material was obtained which does not require conductive additives. The current computational study aims to elucidate the functionality of this material by drawing in‐depth structure–property relationships utilizing a hierarchical molecular and quantum mechanical approach. Initially, structural properties of the electrode material's macroenvironment—containing the functionalized polythiophene, electrolyte, and solvent—were assessed in various charging states by molecular dynamics simulations. Subsequently, electronic properties were investigated by time‐dependent density functional theory for 564 microenvironments. Via this computational setup, the electronic communication within the material was assessed along intrastrand and interstrand CT processes involving the respective TEMPO and polythiophene units. Thereby, our hierarchical computational approach reveals that the intrinsic conductivity and charge storage capacity of the electrode material stems from efficient intrastrand TEMPO‐polythiophene CT processes along short and rigid amid linkers. These insights help to tailor improved conductive organic electrode materials with higher charging and discharging rate capabilities.

## Graphical abstract

Charge transfer processes are investigated in p‐type organic electrode materials utilizing stable organic radicals incorporated in a conjugated backbone. Molecular dynamics simulations and time‐dependent density functional theory calculations allow us to evaluate charge transfer states as well as the underlying structure–property relationships.

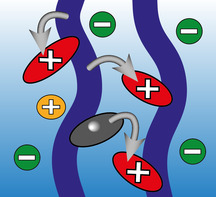

## 1 Introduction

Organic batteries (OBs) are highly promising alternatives and complements to metal‐based energy storage devices. In contrast to metal‐based devices, such as common lithium ion batteries which in case of leakage or physical damage possess potential explosion risks, OBs are nonhazardous [1, 2]. Furthermore, OBs are more environmentally friendly as they do not rely on the exploitation of critical resources and are synthesizable from organic biomasses [3, 4]. Advantageously, such organic energy storage materials can be constructed to be mechanically flexible [5, 6], raising a plethora of possibilities for applications in lightweight devices.

TEMPO, (2,2,6,6‐tetramethylpiperidin‐1‐yl)oxyl, is an extensively characterized p‐type redox‐active material for OBs [7, 8, 9]. The redox‐active moiety of TEMPO is a radical nitroxide group, which is sterically stabilized by four methyl groups. Most often it is utilized in a polymer with a methacrylate backbone, poly(TEMPO methacrylate) (PTMA) [10, 11, 12]. However, materials like PTMA exhibit a flaw in the context of batteries – they lack an intrinsic conductivity. Hence, electrodes based on PTMA require conductive additives such as graphene, carbon black or carbon nanotubes [11]. These additives however reduce the specific capacity as they do not contribute to the charge storage of the electrode.

In contrast, conjugated polymers may be utilized as conductive redox‐active materials and were first introduced as such in the 1980s [13]. However, the semiconductor‐like electronic band structure of conjugated polymers renders their redox‐potentials dependent on their state‐of‐charge [14, 15]. Consequently, charging and discharging capacities as well as voltages of such polymers are unstable [16]. To date, this behavior has circumvented their application as charge‐storage unit in organic batteries.

A new approach is to combine stable radicals with conjugated polymers in order to complement each other's advantages and to compensate for each other's disadvantages [17, 18, 19, 20, 21, 22]. The conjugated polymer forms the backbone of the new hybrid‐material, while the stable radical is attached to the backbone by means of side‐chains. The charge‐storage capacity is then achieved by means of the stable radical unit, while during charging and discharging of the device the charge is transferred from the stable radical to the conducting backbone. This charge localization is necessary to prevent unstable charging and discharging capacities as well as voltages as observed for pure conjugated polymers. Zens et al. postulated four criteria to assess the efficiency of materials within this approach [20]. (I) The backbone should feature a good conductivity. (II) In order to rationalize charge localization on the stable radical, the Gibbs free energy of the charge transfer (CT) process from the radical unit to the backbone should be positive (slightly endergonic). (III) The electronic communication between the stable radical and the backbone should be sufficient, to ensure an efficient CT. (IV) The activation energy of the CT should be small. Except for (III), these criteria compete with each other as the activation energy of the CT from the radical to the backbone depends on the Gibbs free energy of that CT. Previously, Li et al. [21, 22] reported such competition, i.e., with respect to the conductivity of the backbone (I) and the charge localization (II).

In this context, the Lutkenhaus group pioneered and synthesized a p‐type electrode material based on a thiophene‐polymer decorated by pendant TEMPO groups—chemically connected with C_4_‐C_8_ alkyl‐linkers [21, 22]. However, a significantly decreased conductivity compared to pure poly(3‐butylthiophene) was observed and attributed to a dedoping effect introduced by the side‐chain TEMPO groups. This dedoping effect is caused by differences in ionization potentials of thiophene and TEMPO. Thereby, the ionization potential of TEMPO is lower than the ionization potential of polythiophene, so that the positive charge is thermodynamically stabilized on the TEMPO instead of the polythiophene. Hence, at first glance it seems as if the charge localization on the stable radical prevents the required conductivity. However, Li et al. further observed a dependency of the conductivity on the linker connecting the radical moiety and the polythiophene backbone. The lowest conductivity was obtained for the polymer with the longest alkyl‐chain as the linker. Furthermore, differences in charging and discharging rates of the polymers were observed and again the lowest rates were obtained for the polymer with the longest alkyl‐chain as the linker. This was rationalized by an impediment in chain packing caused by the long alkyl‐chains of the linker. We assume that the long TEMPO containing side‐chains affected the chain packing by hampering the dispersive interactions (“*π*‐*π*‐stacking”) between the thiophene‐polymer strands resulting in the decreased conductivity of the later. Zhang et al. likewise synthesized a thiophene‐TEMPO film, but in the solid state, using a C_6_‐alkyl chain [23]. The authors observed that the conductivity of the material decreased exponentially with increase in number of side‐chains and attributed this behavior to fewer crystalline domains of the material caused by steric hindrances by side‐chains impacting the packing of the material. These results indicate that criteria (I) and (III) compete with each other but are not mutually exclusive. Thus, other design concepts might be established to increase the conductivity and to foster charge localization.

**FIGURE 1:**
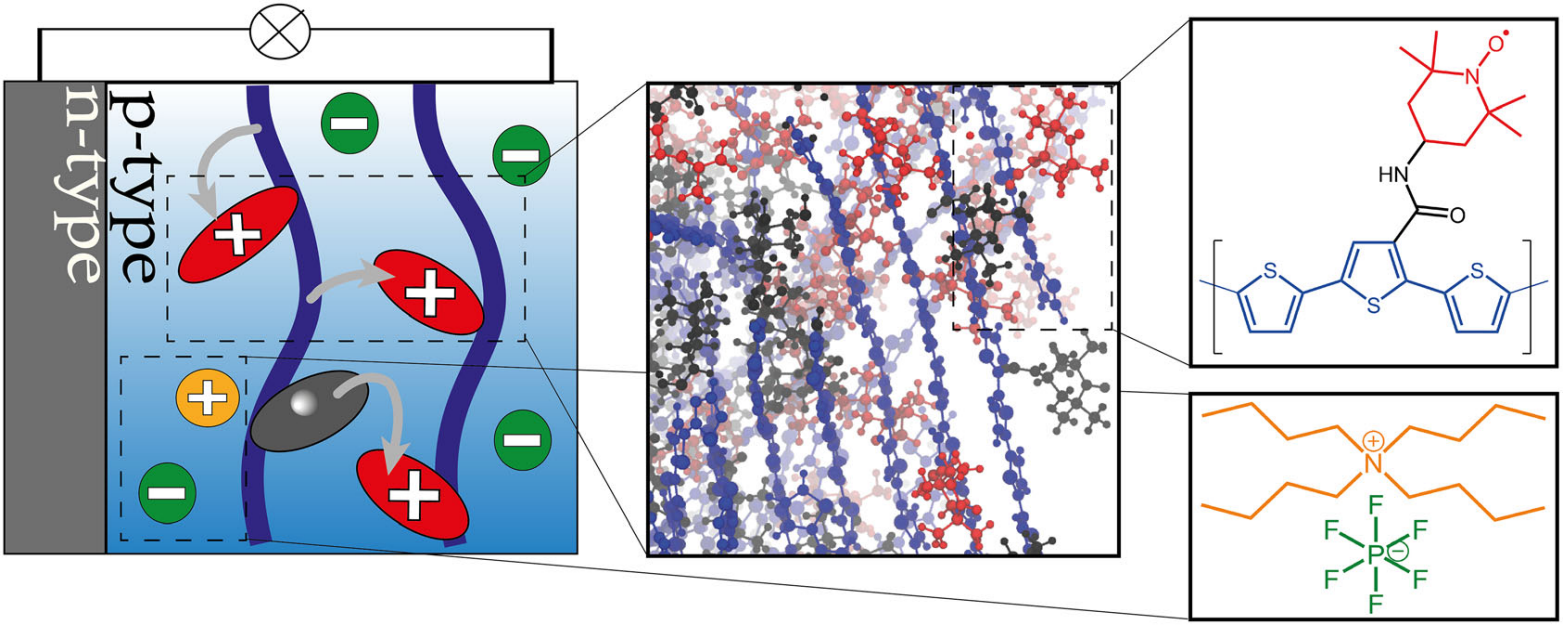
Left: Schematic picture of an organic battery. Blue lines denote the conductive backbone, red denotes the charged TEMPO side‐chains, and gray marks the uncharged TEMPO side‐chains. IntraThio, InterThio, and InterTEMPO are marked with arrows from top to bottom. Middle: Equilibrated MD snapshot. Right: molecular structure of the molecular precursor used as the monomeric subunit of the polymer and the utilized electrolytes.

Very recently, Friebe et al. fabricated a fully functional cell, with a purely organic p‐type electrode based on a polythiophene‐TEMPO polymer, called the proof‐of‐principle (POP) electrode; see Figure 1. To the best of our knowledge, this is the first fabricate of a purely organic functioning electrode without the usage of any conductive additives [19]. In the case of conductive additive‐free n‐type electrode materials, it shall be noted that the Lutkenhaus group also reported a conjugated‐polymer‐based electrode [24]. For the p‐type electrode material introduced by Friebe and coworkers, a short formamide linker was utilized in order to attach the TEMPO units to the thiophene backbone and to provide the necessary electronic communication between the charge‐storage moiety and the conductive aromatic backbone.

The influence of the linker on the functionality of the cell should not be underestimated. It evidently not only affects the packing of the material, but also the CT between TEMPO and thiophene. This is suggested by our precedent computational study, which reported an investigation of the CT processes in a series of molecular precursors with respect to the length and chemical nature of the linker as well as regarding the TEMPO‐polythiophene loading (Figure S1) [20]. It was found that, while the linker was not involved in the CT itself, its chemical—electron‐withdrawing—nature impacted the Gibbs free energy of the CT. Furthermore, the length of the linker correlated negatively with the magnitude of the intramolecular coupling. Thus, the longer the linker, the worse the electronic communication between TEMPO and the backbone.

The observations reported by Li et al. and Zhang et al. depict a material with impeded rate capabilities, while those reported by Friebe et al. depict a material with sufficient rate capabilities. These contrasting observations highlight that further detailed studies on these electrode materials are necessary. For this, an in‐depth understanding of the CT processes is essential to unravel the functionality of redox‐active electrode materials. Such insights are crucial to establish structure–property relationships in order to design efficient and long‐term stable redox‐active organic electrode materials. Of particular interest is elucidating the mechanism of the CT on a molecular level in such OBs and the following questions arise: Is the dedoping effect of TEMPO compensated by another effect in the POP electrode? How does the formamide linker affect the packing and how does this packing influence the CT between TEMPO pairs as well as thiophene? How do these effects intertwine in relation to the four postulated criteria. These insights again might give vital indicators to improved design approaches of related OBs.

To obtain these insights, this fully computational study utilizes molecular dynamics (MD) simulations in order to gain knowledge of the structural properties in such an (cathodic) organic electrode material at a macroenvironmental level. This involves not only the redox‐active polymer but also the electrolyte and solvent. Subsequently, the electronic properties are investigated at the density functional and time‐dependent density functional level of theory (DFT and TD‐DFT) based on key structural properties at a microenvironmental level. Thereby, the setup for the MD simulations strictly follows the chemical design of the POP electrode [19]. In order to evaluate macroenvironmental structural effects (such as packing) and electronic properties of the electrode material and their dependency on the state‐of‐charge (SOC), i.e., at 100%, 50%, and 0% charging. For the semi‐charged system (SOC = 50%), the efficiency of the CT is gauged at the microenvironmental level by the magnitude of electronic communication, or specifically the diabatic coupling. Each microenvironment is comprised of a monomeric unit along the polymer and its environment in a 0.8‐nm radius. These microenvironments shall be called snapshots hereafter.

This procedure of performing quantum chemical calculations in a microenvironment derived from a MD macroenvironment is roughly based on a purely theoretical study by Mitra et al. [25]. There we investigated the impact of orientation and distance between TEMPO–TEMPO pairs onto the underlying electronic coupling, i.e., the electronic communication. This recent study thoroughly evaluated the applicability of different electronic structure methods, e.g., multiconfigurational methods and TD‐DFT, basis set dependencies with respect to minimally augmented double‐zeta and triple‐zeta basis sets, and thus provided us with a robust computational toolkit for the present investigation. Our hierarchical molecular mechanical and quantum chemical approach suggests that our TEMPO‐decorated polythiophene material is organized in semicrystalline structures in contrast to findings regarding similar materials with longer linker‐chains, which presumably disrupt the packing and semicrystalline structure and thereby reduce the (dis)charging rate capability of the material. Furthermore, the present study suggests that CT processes in our material are most efficient within one strand (intrastrand) in contrast to CT processes between different strands (interstrand). Not only is the intrastrand CT more likely to occur compared to interstrand processes but it also features better electronic communication based on the sampled structural parameters.

## 2 Results and Discussion

In order to obtain a comprehensive insight into the functionality of the working p‐type electrode material, first we examine structure properties on a macroenvironmental level allocated by the MD simulations. Subsequently, we shift our computational focus to the microenvironment by analyzing properties of the electronic states involved in the CT processes.

**FIGURE 2:**
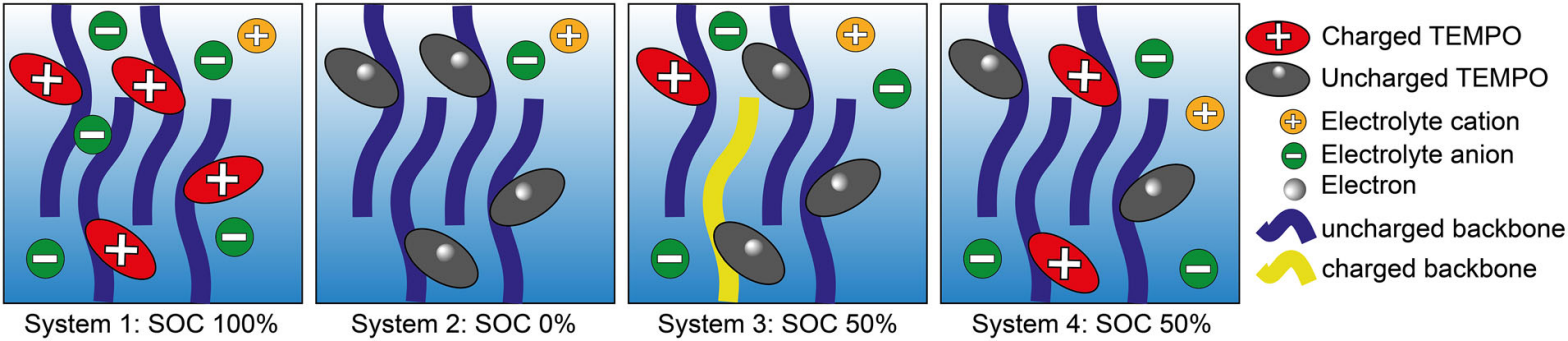
Simplified scheme of the setup of the four systems with states of charges (SOCs) of 100%, 0%, and 50%. Thereby, red ovals denote charged TEMPO moieties, blue lines denote the uncharged thiophene backbone, gray ovals denote uncharged, radical TEMPO moieties, yellow lines denote charged thiophene backbones, green circles denote anionic electrolytes, and orange circles denote cationic electrolytes.

For the MD setup, forcefields for a redox‐active oligomer in three different states of charge (SOC) types were parametrized (see Figure S1 for the monomer and Figure S13 for the oligomer). The first one has the positive charge localized on the nitroxide moiety of the TEMPO (F_NO_). The second one is uncharged (F_u_). The third one has the positive charge localized on the thiophene backbone (F_T_). These oligomers were used to set up four different systems to mimic the electrode material and its underlying structural and electronic properties in various states of charge, i.e., with a SOC of 100%, 50%, and 0%. The first system depicted a SOC of 100% and contained only F_NO_ oligomers. The second system depicted a SOC of 0% and contained only F_u_ oligomers. The third and fourth cells both depicted a SOC of 50% and half of all oligomers were of the uncharged F_u_ type. In the case of the third system, the other half of charged oligomers was made up half by the F_NO_ type and half by the F_T_ type. In the case of the fourth system, the other half of charged F pentamers was made up solely by the F_NO_ type. A simplified scheme of these systems is shown in Figure 2.

All four systems show layering of the thiophene backbones based on dispersive interactions (i.e., *“π*‐*π*‐stacking”) among the (quasi‐)planar polythiophenes with a strand‐to‐strand distances of approximately 3–4 Å. Though the layers are not perfectly stacked, a semi‐crystalline structure is observed. Densities around 1.06 g/cm^3^ and 1.00 g/cm^3^ are obtained for the charged and uncharged systems, respectively, which is a typical density regime for conducting polymers [26].

### 2.1 Structural Properties and Charging

**FIGURE 3:**
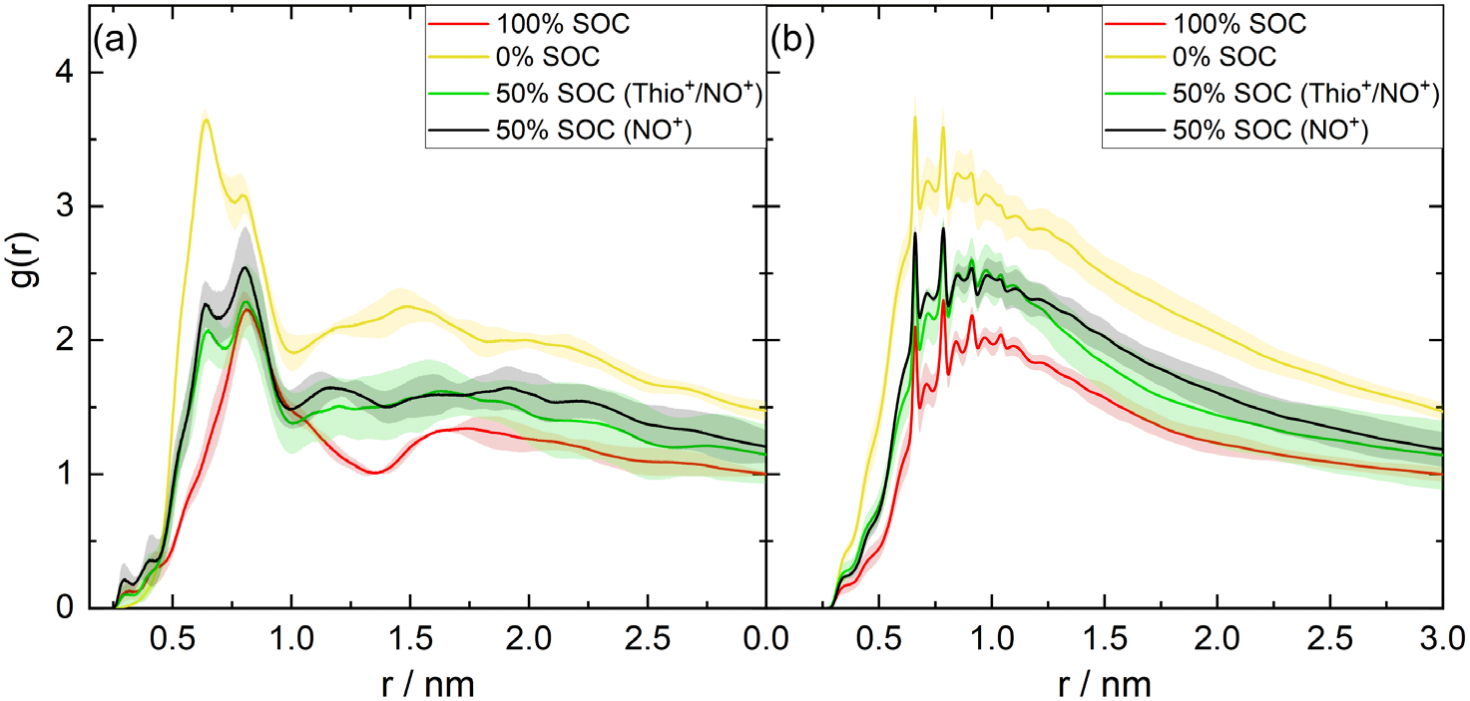
Radial distribution functions for (a) NO–NO and (b) NO‐thiophene distances.

In the following section, the structural properties of the given OB electrode material are investigated based on the performed MD simulations (at 298 K) and depending on the SOC. Thereby, our computational focus was set on examining the distances between the TEMPO units as well as between the TEMPO units and the thiophene moieties. To this aim, radial distribution functions (RDFs) of nitroxyl–nitroxyl distances $g(r_{\mathrm{NO}-\mathrm{NO}})$ and RDFs of the nitroxyl‐thiophene distances $g(r_{\mathrm{NO}-\text{thio}})$ were utilized. The dataset for the analysis stems from the final runs, i.e., production runs, of three independent MD simulations for each system. Averaged RDFs are shown in Figure 3.

Figure 3a depicts RDFs of nitroxyl–nitroxyl distances. To focus on intermolecular nitroxyl–nitroxyl interactions, $g(r_{\mathrm{NO}-\mathrm{NO}})$ is displayed exclusively for distances of 0.15 nm and larger. Notably, a pronounced peak at shorter distances (*r*  =  0.12 nm) reflects the intramolecular N—O bond length. For intermolecular arrangements, two distinct peaks are predicted at 0.64 and 0.80 nm. Both peaks are attributed to interactions of TEMPOs on different polythiophene strands “stacked” together. The two peaks roughly correspond to different TEMPO orientations which are facilitated by rotations around the linker. However, the intensity and peak ratios change according to the SOC. For the uncharged system (0%), both distances contribute to the structural ensemble, while shorter TEMPO–TEMPO distances (0.64 nm) are slightly more frequent. Upon increasing the SOC to 50%, the peak intensity and thus the frequency of larger TEMPO–TEMPO distances increases. This finding accounts for the increasing Coulombic repulsion of the partially charged nitroxyl moieties. In the fully charged state (100%), the TEMPO–TEMPO arrangement at 0.64 nm is merely visible as a shoulder and the dominating structural motif among neighboring TEMPO groups is shifted in favor of larger distances (0.80 nm), which again relates to the NO^+^‐NO^+^ repulsion.

In a similar fashion, RDFs reflecting distances between the nitroxyl moieties and the thiophene backbone were analyzed; see Figure 3b. Significant peaks are observed for various TEMPO‐thiophene distances below 1 nm, namely at 0.66, 0.71, 0.78, 0.85, and 0.91 nm. The even distribution of peaks at distances of 0.6–0.7 nm likewise indicates crystalline domains within the TEMPO‐thiophene material. Notably, the shape of the RDFs does not significantly vary depending on the SOC. This finding suggests that the overall crystalline‐like packing of the stacked thiophene backbone strands is preserved upon charging and discharging. Different overall magnitudes of the gyration radii in different cells are attributed to different normalization factors due to different average particle densities in the cells, depending on the SOC.

RDFs of $g(r_{\mathrm{NO}-\text{thio}})$ and their dependency on the SOC imply that no significant structural rearrangement of the polythiophene backbone occurs upon charging (discharging) of electrode material. In contrast, $g(r_{\mathrm{NO}-\mathrm{NO}})$ implies that neighboring TEMPO moieties undergo structural rearrangement based on the static (repulsive) interaction upon charging. The structural preservation of the polythiophene backbone and the simultaneous structural rearrangement of TEMPO moieties are mediated by the flexible amide linker which allows larger TEMPO–TEMPO distances upon charging while keeping the backbone structure almost unaffected.

A similar structural rearrangement of charge‐storage moieties with increasing SOC was previously reported in a nonconductive redox‐active polymer [27]. Due to the repulsive interactions between the redox‐active moieties, the CT among each other was impeded. This phenomenon is circumvented in the POP electrode material as the redox‐active moieties involved in the CT do not only include the charge‐storage moieties but also the conducting backbone which we find unaffected by the SOC.

The RDFs further show no significant differences between the two simulation cells with SOC = 50% (TEMPO and mixed TEMPO‐thiophene charging). As a positively charged thiophene backbone is thermodynamically unfavorable with respect to a positively charged TEMPO, further quantum chemical calculations are based on the simulations with SOC = 50% with charge only localized on the TEMPO units.

### 2.2 Intrastrand vs. Interstrand Charge Transfer

**FIGURE 4:**
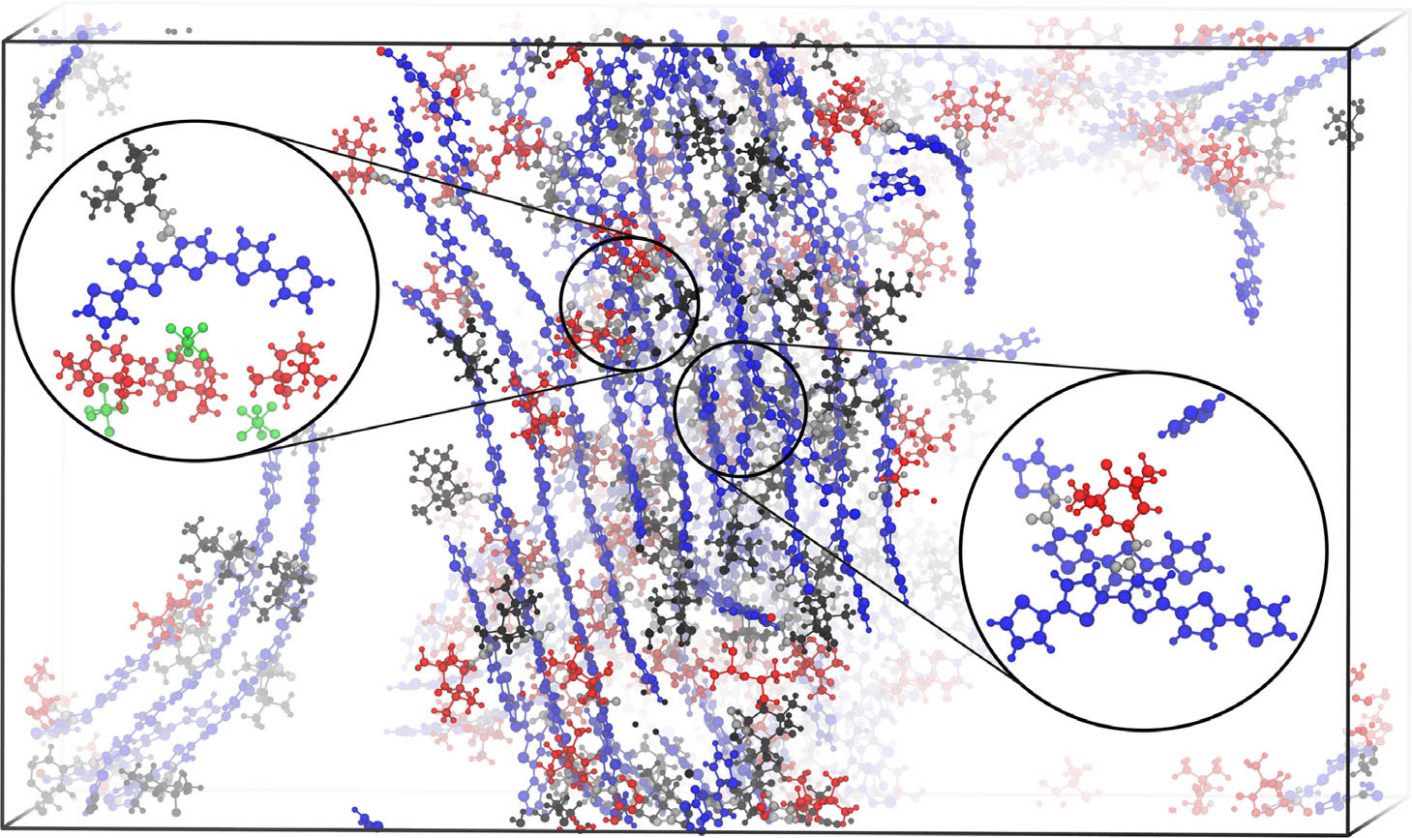
Representative structure of the second production run of the fourth system with SOC = 50% and charge only localized on TEMPO. The uncharged thiophene backbone is shown in blue, the chemical linker in silver, charged TEMPOs in red, and uncharged TEMPOs in black. Semi‐crystalline regions are seen along the alignment of the blue‐colored backbones. Zoomed in are two exemplary snapshot geometries with the same color code as for the whole box; additionally, PF_6_^−^ is shown in green.

In the following section, our computational focus was set on evaluating the electronic properties in the microenvironment of the p‐type electrode material. To this aim, the microenvironment was described by means of snapshots taken from the MD simulations of the fourth system, with a SOC of 50% and the charge localized on the TEMPO moieties (Figure 4). These snapshots were obtained by iterating over each monomeric unit along each oligomer in the MD system and including every thiophene or TEMPO unit within a radius of 0.8 nm from the TEMPO moiety of the respective monomeric unit. The cutoff distance of 0.8 nm was chosen as our previous computational benchmark study [25] revealed that couplings for long‐range CT, e.g., beyond 0.8 nm are negligible. Consequently, the electronic communication and thus the charge transfer efficiency is limited to neighboring TEMPO moieties. Furthermore, RDFs for the NO–NO distance (Figure 3a) indicate the highest probability of finding one NO next to another NO at distances smaller than 0.8 nm. Snapshots comprising more than 200 atoms were split into smaller snapshots.

**FIGURE 5:**
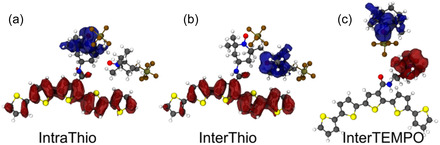
Attachment (blue) and detachment (red) densities of charge transfer processes in different snapshots to exemplary show (a) IntraThio, (b) InterThio, and (c) InterTEMPO CT types.

Snapshots were taken at the beginning and the end of each of three 20‐ns production runs. Hence, a total number of 851 snapshot geometries was retrieved. It shall be noted that while the monomeric subunits within the snapshots are the same at each time point, the surrounding environment is updated at each time point according to the described evaluation pattern. Hence, snapshots at *t* = 20 ns do not necessarily represent the same geometries as the snapshots at *t* = 0 ns. On each of these snapshots, TD‐DFT calculations were performed to cover both intra‐ and interstrand electron transfer processes of NO^•^ → NO^+^ and thio → NO^+^ character. Thereby, intrastrand CT of thio → NO^+^ character is denoted IntraThio, interstrand CT of thio → NO^+^ character is labeled InterThio, and interstrand CT of NO^•^ → NO^+^ as InterTEMPO, respectively (see Figure 5). Notably, the number of (excited) states was adjusted dynamically with respect to the system size as several locally excited states of the polythiophene are found energetically in between the energy range given by the charge transfer states of interest. Subsequently, adiabatic CT states between the thiophene backbone or/and between TEMPO (pairs) were identified in an automated fashion and diabatized to obtain the potential couplings by means of Edmiston–Ruedenberg diabatization as implemented very recently in our program suite pysisyphus [28]. This way, the electronic communication between *n*‐electronic states (potential couplings) can be obtained in a straightforward fashion without the need for dipole moments (e.g., generalized Mulliken−Hush approach [29]) or charge localizations (fragment charge difference [30, 31]) for the involved electronic states. Of the 840 snapshot TDDFT calculations, 9 failed due to convergence issues. 267 snapshots failed in the adiabatic state sorting/diabatization procedure, as no suitable adiabatic states were found for diabatization. Finally, diabatic states and couplings for 564 snapshots were obtained. A more detailed description of the overall procedure is presented in the computational details section.

**FIGURE 6:**
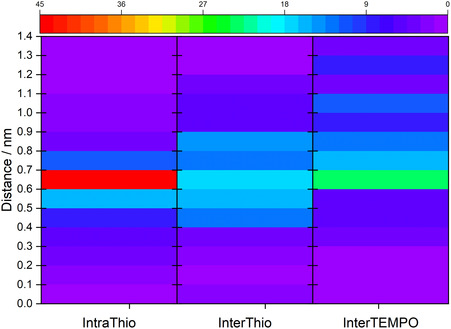
Occurrence of charge transfer (CT) processes attributed to the interstrand TEMPO–TEMPO (InterTEMPO) charge transfer (CT) type, the interstrand TEMPO‐thiophene (InterThio) CT type, and the intrastrand TEMPO‐thiophene (IntraThio) type. Occurrences of CTs are plotted relative to the distances between donor and acceptor group of the CT. Occurrences are indicated as percentages relative to all occurrences of CTs within InterTEMPO, InterThio, and IntraThio. Percentages are indicated as a heatmap in the color range from purple to red, where purple denotes no CTs and red 45 CTs.

Herein, the focus of our analysis is shifted to the distance dependency of the abovementioned CT processes to examine structural effects on the CT on a molecular scale. Distances between donor and acceptor groups were evaluated based on their adiabatic excited states’ detachment and attachment densities. Thereby, the smallest distance between atoms on which these densities were located was evaluated. The heatmap for the occurrence of a type of CT within a certain distance is illustrated in Figure 6.

In case of the IntraThio CT, the overall magnitude of CTs is observed at distances between 0.6 and 0.7 nm. This distance range corresponds to the distance between the nitroxyl‐moiety of TEMPO and the (nearest) carbon atom of the thiophene‐backbone to which the linker is attached. For the intermolecular CT types, these distance ranges are broader and shifted to larger distances. Thereby, a higher proportion of InterTEMPO CTs is observed at distances above 0.8 nm compared to InterThio CTs. These narrower distance distributions of the IntraThio CTs compared to the interstrand CTs derive from geometric constraints by the linker, which are not restricting the interstrand CTs processes.

For CTs with strong couplings above 10 meV, a similar distance distribution as for all CTs is found. Thus, for the IntraThio CT, a narrow distribution is found where most CTs with a coupling above 10 meV are found at distances between 0.6 and 0.8 nm, while interstrand CTs display a broader distance distribution of CTs with couplings above 10 meV. That these broad distance distributions are also observed for higher couplings demonstrate that the donor–acceptor distance is not the major factor contributing to the magnitude of the potential coupling. As emphasized in our previous study on TEMPO–TEMPO^+^ pairs [25], the general orientation of the donor and the acceptor likewise affects the magnitude of the potential coupling to a great extent. However, a detailed analysis of the orientation for inter‐ and intrastrand CTs is beyond the scope of this article and will be conducted in a subsequent study.

**FIGURE 7:**
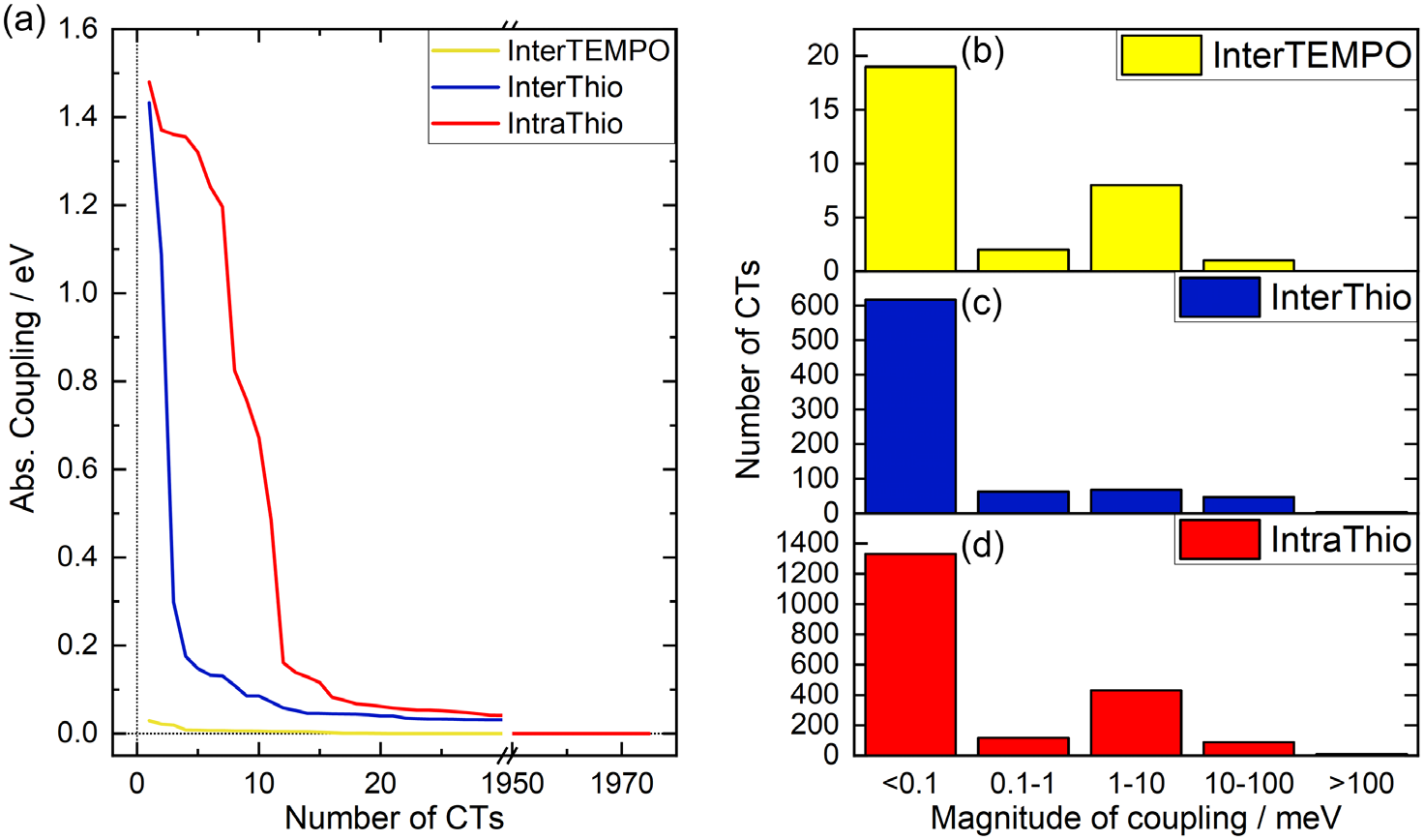
(a) Absolute diabatic coupling per charge transfer process sorted by magnitude. Yellow denotes couplings for interstrand TEMPO–TEMPO (InterTEMPO) charge transfer (CT) processes, blue denotes couplings for interstrand TEMPO‐thiophene (InterThio) CTs, and red denotes couplings for intrastrand TEMPO‐thiophene (IntraThio) CTs. (b–d) Number of CT processes within ranges of magnitude of coupling of <0.1 meV, 0.1–1 meV, 1–10 meV, 10–100 meV, and >100 meV for (b) InterTEMPO, (c) InterThio, and (d) IntraThio.

Figure 7a illustrates the absolute coupling in eV for the first 30 CTs sorted by magnitude of coupling. Figure 7b–d illustrates the amount of CT processes in a given coupling range for the three different CT types, InterTEMPO, InterThio, and IntraThio.

It should be noted that due to the snapshot‐generation procedure, which could not utilize periodic boundary conditions, the number of snapshots containing only one monomeric unit is overestimated. Hence, very likely also the number of IntraThio CTs is overestimated compared to the number of interstrand CTs. As a result, occurrences of CT types shall only be discussed on a qualitative level. However, magnitudes of couplings are still discussed on a quantitative level.

Prior to the in‐depth discussion on the CT processes of interest, it shall be noted that intra‐ and interstrand thiophene–thiophene CT types, i.e., the formerly mentioned locally excited π‐π* states of the polythiophene, are by far the most frequent processes in all snapshots. These states reflect the intrinsic conductivity of the aromatic backbone, and hence, their frequency indicates a good conductivity and fulfillment of the first abovementioned criterion. However, as these states are not directly involved in the CT between the redox‐active units and the backbone, they are not taken into account for further analysis.

Based on this setup, a total number of 2881 CT processes were investigated. Among these processes, InterTempo occurs in only 1% of cases. This low contribution of TEMPO–TEMPO CTs compared to TEMPO‐thiophene CTs is an interesting phenomenon as the RDFs (Figure 3) show rather similar separation distances between nitroxyl moieties as between nitroxyl moieties and the thiophene backbone. We hypothesize two main reasons for that phenomenon. Firstly, Figure S12 reveals that the highest contribution of TEMPO–TEMPO CTs is in the adiabatic state energy range of 2.8–3.0 eV suggesting that above that excitation energy there might be more contributions of TEMPO–TEMPO CTs. In the present computational setup, we utilize an energy cutoff for the diabatization of 3 eV as higher lying CT processes are thermodynamically unfeasible. Secondly, TEMPO's methyl groups shield the nitroxide moieties. On the one hand, this effect stabilizes the radical unit; on the other hand, it also inhibits the CT from one TEMPO to the other (also shielded) TEMPO units. In case of the TEMPO‐thiophene CTs, the methyl groups of the TEMPO also partially hamper the CT; however, “double‐shielding” as in InterTEMPO is not present due to the extended *π*‐system of the thiophene‐backbone. However, a detailed investigation into this effect is beyond the scope of the present manuscript and will be carried out in a subsequent study.

In addition to InterTEMPO‐type CTs being infrequent, also two thirds of CTs of this type exhibit a coupling that is smaller than 0.1 meV. These conditions render the InterTEMPO CT very inefficient and raise the need for the conductive backbone or, otherwise, conductive additives. It should be noted that in our precedent study on the electronic communication in TEMPO–TEMPO pairs [25], calculated potential couplings were in a similar magnitude range as in our current study. However, in our previous study, a much higher percentage of CTs featured couplings in the range of 1–100 meV. This divergence in distribution of potential couplings is attributed to the fact that our previous study considered merely TEMPO without any environmental effects. In contrast, our current investigation pursues a slightly more realistic approach and includes (implicit) solvent effects as well as surrounding molecules changing the electronic environment of the TEMPO–TEMPO pairs (and also thiophene‐TEMPO pairs) and thereby affecting the potential coupling.

Interstrand CT processes between TEMPO and thiophene occur much more often than the CTs between TEMPO units. Within the InterThio CT type, the majority (77%) of potential couplings amount to less than 0.1 meV, while 16% are larger than 1 meV.

Finally, IntraThio CT‐type processes are the most frequently occurring CT phenomena, of which 67% feature a diabatic coupling of less than 0.1 meV. These very weak intramolecular couplings (obtained by Edmiston–Ruedenberg diabatization) are in accordance with the couplings obtained in our computational study on potential monomers [20]. In this previous article, the (intramolecular) coupling for the respective monomer was calculated as 0.03 meV by means of the minimum energy splitting approach as well as using the fragment charge difference method. In this study, the timescale of the CT was gauged to be 52 ms by means of Marcus theory. Assuming that the Gibbs free energy and the reorganization energy of the here examined IntraThio CTs are in a similar range as for the previously examined monomer, the CTs with couplings of <0.1 meV would be slower than the rearrangement of molecules observed within this MD simulation, decreasing the probability of these CTs drastically. A smaller fraction of 26% of the IntraThio CTs feature significantly stronger potential couplings in the range of 1–10 meV, which would translate to charge transfer kinetics in the range of picoseconds—based on our previously evaluated semi‐classical Marcus CT kinetics (and dissipative quantum dynamics) in the respective molecular precursor [20]. Interestingly, there are significantly more occurrences of couplings in the range of 1–10 meV than in the range of 0.1–1 meV. This phenomenon is consistent for all evaluated time points of all three production runs. Furthermore, this phenomenon is only observed for the evaluated intrastrand CTs not for the interstrand CTs, which suggests that geometric constraints play a major role in the efficiency of the electronic communication.

Notably, Kemper and colleagues published a series of studies where they investigated CT properties in PTMA [12, 32, 33], where they found among other things that interstrand CT from head‐on and face‐to‐face arrangements of TEMPOs were the main contributors to the CT [32]. This is likely attributed to the nonconductive backbone which constrains arrangements between intrastrand TEMPOs. Contrastingly, in the case of the herein reported POP redox‐active polymer, intrastrand CT is frequently observed, due to the redox‐active nature of the backbone enabling it to take part in the CT itself and the presence of a linker which keeps the redox‐active moieties within close proximity to each other. This contrasts with the Lutkenhaus electrode material [22], where we believe that interstrand CTs are mostly prevalent due to the long alkyl chains which diminish the IntraThio CT as a result of a broad structural distribution and long intrastrand distances (resulting in potential coiling and shielding) between the redox‐active moieties.

Similar to the electronic coupling, the adiabatic excitation energy may give insights into how likely a CT is. Figure S12 visualizes the frequency of adiabatic excitation energies sectioned into adiabatic IntraThio, InterThio, and InterTEMPO CT types. While for the intermolecular CT types there is a rather broad distribution of excitation energies above 1.0 eV, an interesting trend is observed for the IntraThio CT type. Two distinct accumulations of excitation energy are visible in the energy windows of 1.6–2.2 eV and 2.6–3.0 eV; any excitation energies above 3.0 eV were not considered in this study. These accumulations do not correlate with the accumulations of electronic couplings nor with the distance. We attribute these accumulations of excitation energy in the IntraThio type to the structural prearrangement due to the chemical linker. The broad distribution in excitation energies for the interstrand CTs derives from a much wider structural arrangement between thiophene and TEMPO or TEMPO–TEMPO pairs.

### 2.3 From Computation Modeling to Electrode Material Design Concepts

These results give insights into how the POP electrode is characterized in regard to three of the four postulated criteria outlined above.

#### 2.3.1 (I) Conductivity and (II) Charge Localization

As pointed out in the introduction, Li et al. observed low conductivities in their thiophene‐TEMPO material and attributed this to two main reasons: firstly, an amorphous material due to the long alkyl linkers and, secondly, a dedoping effect of the nitroxyl moieties. Our MD simulation shows dispersive interactions or “*π*‐*π*‐stacking” (3–4 Å) between the thiophene backbones leading to semi‐crystalline structure of the electrode material. The crystallinity of organic conducting polymers is known to have a high impact on the conductivity of the material. The more crystalline the material, the better the charge‐conducting properties are. At the same time, the electrolyte diffusion properties decrease with increasing crystallinity. Hence, the semi‐crystalline packing of the material in the POP electrode might enhance the conductivity along the thiophene backbone while at the same time allowing good electrolyte diffusion. The latter, however, was not evaluated in the present study and requires significantly longer production runs up to the µs‐regime.

Quantum mechanical analyses reveal that the positive charge in the ground state is localized on the nitroxyl moiety—independent of the geometry and the surrounding environment. Thus, stable charging and discharging voltages and capacities are provided. This charge localization indicates a similar dedoping effect as observed by Li et al. However, we hypothesize that due to the semi‐crystalline packing of the cell this stable charge localization does not hamper the conductivity.

While we cannot make any further assertions regarding the conductivity and doping of our POP material based on our current results, the previous study by Friebe et al. suggests that the electrolyte has a significant influence on the performance of the electrode material. Whether this impact stems from a chemical doping mechanism or otherwise will be the subject of future investigations.

#### 2.3.2 (III) Electronic Communication

The results of this study reveal that in the majority of snapshots where a CT could happen, the coupling is so low that the CT is kinetically unfavorable and, hence, is very inefficient. This phenomenon is independent of the type of CT. Furthermore, we found that the likelihood of electronic couplings above 1 meV is increased for the IntraThio CT type compared to the interstrand CT types. Couplings at this magnitude and above render the kinetics of the CT much faster; the respective rate constant scales with the square of the electronic coupling. Hence, the CT efficiency is increased tremendously. We attribute geometric constraints in the IntraThio CT to the increased magnitude in couplings; however, the precise reason for this needs further elucidation. Another advantage of the IntraThio CT type compared to its intermolecular pendants is that donor and acceptor units are always in proximity to each other. While there are many snapshots where no intermolecular CT was observed at all, the IntraThio CT was almost always observable. Hence, not only the likelihood of finding high couplings but also of finding the CT itself is increased for the IntraThio CT types compared to the intermolecular CT types.

## 3 Conclusion and Outlook

In this fully computational contribution, competitive charge transfer processes in a p‐type electrode material for application in organic batteries were systematically investigated. The studied electrode material, very recently introduced by the Schubert group [19, 20], is built from noncrosslinked polythiophene strands and is decorated (using formamid linkers) with redox‐active TEMPO units in combination with a Bu_4_NPF_6_ electrolyte. Thereby, TEMPO functions as the charge‐storage unit(s) while the aromatic backbone provides the intrinsic conductivity. Consequently, no additional conductive additives are required.

The focus of our theoretical investigation is set on identifying macromolecular structural properties and evaluating CT processes affected by these structural properties. These CT processes were analyzed in regard to their electronic communication. Thereby, CT processes were divided into intrastrand and interstrand CT processes between thiophene and TEMPO as well as TEMPO pairs. Thereby, the intrastrand CT is enabled by the chemical linkage of the charge‐storing TEMPO units to the charge‐conducting polythiophene backbone. This linker introduces geometric constraints between the charge storage and charge conductor unit such as distance and orientation of both units toward each other. It was found that the intrastrand TEMPO‐thiophene CT is dominating the redox processes associated with the charging and discharging of the p‐type electrode material. The intrastrand processes occur more frequently in comparison to interstrand CT phenomena and provide a better electronic communication between the components (as given by the potential coupling matrix elements). Based on our precedent study on the respective molecular precursors and in combination with synthetic as well as cell studies, we suggest that this intramolecular CT is favorable for short linkers—ideally C_1_‐linkers—which connect the TEMPO unit to the (oligo)thiophene. However, in order to maintain the semi‐crystalline organization of thiophene also at a SOC = 100%, the linker should still possess a level of flexibility, namely, be sufficiently flexible as to allow charged TEMPO species to evade each other without affecting the integral structure of the backbone.

The findings of our study are based on our “proof‐of‐principle” electrode, but may be transferred to other p‐type materials with a conducting backbone and charge‐storing side‐chains. Hence, our findings allow for targeted improvements in these design approaches. Thereby, the key insight of this study toward a better electrode design is that short linkers between charge‐storage and charge‐conductor unit improve the functionality of the cell. This finding suggests that crosslinkers might improve the functionality of the cell even further. Thus, future studies shall examine the effect of crosslinkers onto the CT properties of these materials. Last but not least, the mechanism behind the impact of geometric constraints introduced by the linker onto the electronic communication deserves another detailed examination.

## 4 Computational Details

This study examined an oligomer consisting of five monomer units based on a TEMPO unit linked by formamide substituent to a terthiophene backbone described in Friebe et al. [19] and Zens et al. [20] (Figure S13). This oligomer will hereafter be called the F pentamer. Molecular dynamics simulations were performed with Gromacs [34] using the OPLS‐AA [35, 36] force field. Gromacs was packaged by the Nix package manager [37]. Three differently charged F pentamers were used for simulation in order to approximate the polymer state in an actual battery.

1. The positive charge was localized on the nitroxide moiety (F_NO_)
2. The polymer was uncharged (F_u_)
3. The positive charge was localized on the thiophene backbone (F_T_)

Force field parametrization of the acetonitrile (ACN) as solvent and Bu_4_N^+^ and PF_6_^−^ as electrolytes as well as the F pentamers was conducted as follows:

All molecular structures were optimized in Gaussian16 [38] at the *ω*b97xd [39]/def‐svp [40] and reoptimized on the *ω*b97xd/6‐311g(3d2f) level of theory. CM5 [41] charges for the force field parameters were obtained at the latter level of theory. CM5 charges were chosen instead of the more commonly used ChelpG or RESP charges as they were shown to exhibit a more consistent behavior [42, 43], especially as ChelpG charges have shown an unreasonable dependency of the sulfur charge on the molecular conformation [44].

In the case of ACN, Bu_4_N^+^, and PF_6_^−^, all other force field parameters were obtained with the program mktop [45], which uses already existing force field parameters.

Force field parameters for the F pentamers were obtained in the style described by Chen et al. [46]. To “polymerize” the F monomer, the monomer was optimized but with a conjugated backbone of the length of five thiophenes instead of three. This was done in order to gain the force field parameters of the thiophene dihedrals at the conjunction of the monomers. Three different monomers were optimized in accordance with the three different polymers described above. Force fields for the monomers were obtained with the program LigParGen (online) [36, 47, 48]. CM1A charges from LigParGen's force field parameters were replaced with the Gaussian calculated CM5 charges. For uncharged systems, Dodda et al. suggest to apply a scaling factor of 1.14 to the CM5 charges [49]; however, as two of the three monomer kinds are charged, no scaling factor was applied to any system to be consistent.

PDB files of the polymer strands were obtained by aligning five monomers with the help of the additional thiophenes at the ends of the monomer backbone along with each other. Force field parameter files were concatenated with a Python script; double entries originating from the additional thiophene rings at the monomer backbone were erased.

Simulation cells of the size of 12 × 7 × 7 nm^3^ were packed with packmol [50]. Thereby, 354 molecules of Bu_4_N^+^ and 354 (+ number of pos. charges of the F pentamer) molecules of PF_6_^−^ were used in order to gain an electrolyte concentration of 1 mol L^−1^. Based on experimental findings, the number of F monomers in such a cell would lie between 112 and 1967. Hence, as a start approximation, 22 F pentamers were packed in a cell with packmol, and the amount of pentamers was increased until packmol would fail to converge. In this way, a maximum amount of 28 F pentamers was obtained and used in all simulations. The solvent acetonitrile was added with the module *gmx solvate*.

Four different simulation cells were packed this way. The first consisted only of F_NO_ pentamers and depicts a SOC of 100%. The second consisted only of F_u_ pentamers and depicts a SOC of 0%. The third consisted of 7 F_NO_, 14 F_u_, and 7 F_T_ pentamers and depicts a SOC of 50% as well as the fourth cell, which consisted of 14 F_NO_ and 14 F_u_ pentamers.

Simulations were carried out in a four‐step procedure. Firstly, an energy minimization with the steepest descent algorithm was performed to get rid of any too closely lying atoms. Secondly, the system was relaxed for 25 ns in an NVT‐ensemble with periodic boundary conditions. Time steps of 0.5 ps and the V‐rescale thermostat ($\tau_{T}=1$) were chosen. A heat‐cool cycle loosely based on the procedure of Kemper et al. [12] was performed in order to gain the minimum energy configuration of the system. Thereby, the system was heated up to 700 K, i.e., heated for 5 ns at 700 K, cooled down to 100 K within 1 ns, cooled at 100 K for 100 ps, and afterwards heated up again to 700 K within 1 ns. This procedure was repeated three times; after the last cool‐down cycle, the system was heated up to 298 K. The system was then equilibrated as an NPT‐ensemble for 10 ns with the C‐rescale barostat ($\tau_{p}=2$, *p* = 1.0 bar) and a V‐rescale thermostat ($\tau_{T}=1$, *T* = 298 K). Subsequently, the system was re‐equilibrated with the Parrinello–Rahman barostat ($\tau_{p}=5$) and the Nose–Hoover thermostat ($\tau_{T}=2$) for 10 ns followed by a 20‐ns production run. Each simulation was repeated three times. Radial distribution functions (RDFs) were calculated with the *gmx rdf* module of GROMACS.

Snapshots for analysis were obtained with a Python script making use of the program MDAnalysis [51, 52]. To obtain these snapshots, it was iterated over each monomeric subunit of the 28 F pentamers. Thereby, each monomeric subunit had a backbone of five thiophenes. Five thiophenes were found to be a good compromise in size of the fragment and status of convergence of the ionization potential [20]. As for the diabatic coupling of the charge transfer processes of interest, a volume element around each monomeric subunit was evaluated for “CT counterparts” of the monomeric subunit. Hence, if the monomeric subunit had the charge localized on the TEMPO, every uncharged thiophene ring and every uncharged TEMPO where the nitroxide group was within a distance of 0.8 nm [25] of the charged nitroxide group was included as well as every TEMPO with a charged nitroxide group and every charged thiophene ring within 0.4 nm of the uncharged thiophene backbone. In order to neutralize the snapshots, PF_6_^−^ ions were included with a scheme that adapted the “inclusion distance” depending on the charge of the snapshot. If the snapshot geometry comprised more than 200 atoms, the snapshot was split into two snapshots. The splitting was performed along the thiophene backbone of the monomeric subunit. It was made sure that in both snapshots whole molecules were included. The monomeric subunit was added to both new snapshots.

These selections were “taken” at the beginning and the end of the 20‐ns production run, i.e., 831 snapshots were obtained. In order to fill “cuts” at bonds OpenBabel [53] 2.4 was utilized with slightly changed double bond angle settings, in order to minimize the amount of “hydrolyzed” thiophene double bonds. This version of openbabel was packaged with the Nix package manager [37].

For each snapshot, TD‐DFT calculations were performed with orca 5 [54, 55], packaged with the Nix package manager [37]. The *ω*b97XD3 [56] functional and the basis set ma‐def2‐svp [57] were utilized. Attachment and detachment densities were calculated with the program suite pysisyphus [28]. These were used to determine the nature of the excited state in an automated fashion. Python scripts to determine the nature of the excited state were partially written with assistance of artificial intelligence. If an excited state involved attachment or detachment densities localized on the nitroxide moiety or the thiophene backbone above a certain threshold, it was included into the list of excited states of interest. The respective excited state was excluded again, if the attachment and detachment densities were either localized both on the thiophene backbone or were localized both on the same nitroxide group. Based on the polymer strand where the attachment/detachment densities were localized, the excited states were categorized into inter‐ and intrastrand CT processes. A snapshot was not included if the automated filtering mechanism did not find any fitting excited states. Thus, in total the diabatization was performed for 564 snapshots.

After the excited states of interest were extracted, an Edmiston–Ruedenberg diabatization, as implemented in pysisyphus, was performed to obtain the potential couplings for each diabatic state. Afterwards, again an analysis of the diabatic states was performed, likewise to the analysis of the adiabatic states, categorizing the diabatic states and couplings into IntraThio, InterThio, and InterTEMPO. It shall be noted that the character of states changes after diabatization, so that adiabatic and diabatic categorizations of states are not necessarily the same. Categorizations used within this manuscript in reference to couplings are always diabatic state categories.

## Supporting Information

Additional supporting information can be found online in the Supporting Information section. See the supplementary material for details on potential couplings per category in general and for every single production run and evaluated time step. All trajectories, snapshot geometries, and force field parameters are available free of charge by the Zenodo repository; see Ref [58]. **Supporting Fig. S1:** Investigated monomers from our previous study. TEMPO moieties are shown in red, the bithiophene and terthiophene backbone is shown in blue, the linker is shown in black. The monomer that served as basis for the herein investigated polymer is highlighted in red. **Supporting Fig. S2:** Electronic coupling of snapshots at their respective distances. For better visibility a logarithmic scale was chosen for the couplings. **Supporting Fig. S3:** Evaluated charge transfer (CT) processes in absolute numbers sectioned into intramolecular TEMPO‐thiophene (IntraThio) CTs, intermolecular TEMPO‐thiophene (InterThio) CTs and intermolecular TEMPO‐TEMPO CTs (InterTEMPO). **Supporting Fig. S4:** Evaluated charge transfer (CT) processes in percent sectioned into intramolecular TEMPO‐thiophene (IntraThio) CTs, intermolecular TEMPO‐thiophene (InterThio) CTs and intermolecular TEMPO‐TEMPO CTs (InterTEMPO). **Supporting Fig. S5:** Frequency and magnitude of couplings for intrastrand TEMPO‐thiophene (IntraThio), interstrand TEMPO‐thiophene (InterThio) and interstrand TEMPO‐TEMPO (InterTEMPO) charge transfer processes for all evaluated snapshots. Colors of the heat map indicate the frequency of a specific type of transfer within one snapshot. **Supporting Fig. S6:** Frequency and magnitude of couplings for intramolecular TEMPO‐thiophene, intermolecular TEMPO‐thiophene and intermolecular TEMPO‐TEMPO charge transfer processes for different snapshots taken at *t* = 0 ns of the first production run. Colors of the heat map indicate the frequency of a specific type of transfer within one snapshot. **Supporting Fig. S7:** Frequency and magnitude of couplings for intramolecular TEMPO‐thiophene, intermolecular TEMPO‐thiophene and intermolecular TEMPO‐TEMPO charge transfer processes for different snapshots taken at *t* = 20 ns of the first production run. Colors of the heat map indicate the frequency of a specific type of transfer within one snapshot. **Supporting Fig. S8:** Frequency and magnitude of couplings for intramolecular TEMPO‐thiophene, intermolecular TEMPO‐thiophene and intermolecular TEMPO‐TEMPO charge transfer processes for different snapshots taken at *t* = 0 ns of the second production run. Colors of the heat map indicate the frequency of a specific type of transfer within one snapshot. **Supporting Fig. S9:** Frequency and magnitude of couplings for intramolecular TEMPO‐thiophene, intermolecular TEMPO‐thiophene and intermolecular TEMPO‐TEMPO charge transfer processes for different snapshots taken at *t* = 20 ns of the second production run. Colors of the heat map indicate the frequency of a specific type of transfer within one snapshot. **Supporting Fig. S10:** Frequency and magnitude of couplings for intramolecular TEMPO‐thiophene, intermolecular TEMPO‐thiophene and intermolecular TEMPO‐TEMPO charge transfer processes for different snapshots taken at *t* = 0 ns of the third production run. Colors of the heat map indicate the frequency of a specific type of transfer within one snapshot. **Supporting Fig. S11:** Frequency and magnitude of couplings for intramolecular TEMPO‐thiophene, intermolecular TEMPO‐thiophene and intermolecular TEMPO‐TEMPO charge transfer processes for different snapshots taken at *t* = 20 ns of the third production run. Colors of the heat map indicate the frequency of a specific type of transfer within one snapshot. **Supporting Fig. S12:** Occurrence of charge transfer (CT) processes attributed to the interstrand TEMPO‐TEMPO (InterTEMPO) charge transfer (CT) type, the interstrand TEMPO‐thiophene (InterThio) CT type and the intrastrand TEMPO‐thiophene (IntraThio) type. Occurences of CTs are plotted relative to the respective adiabatic excitation energies of the CT. Occurences are indicated as absolute numbers represented as a heatmap in the color range from purple to red, where purple denotes no CTs and red 310 CTs. **Supporting Fig. S13:** Picture of one polymer strand of F_NO_.

## Funding

This work was supported by Deutsche Forschungsgemeinschaft (Grand 441265816).

## Conflicts of Interest

The authors declare no conflicts of interest.

## Supporting information

Supplementary Material

## Data Availability

The data that support the findings of this study are openly available in [Zenodo] at [10.5281/zenodo.17628566], reference number [58].
