# Supplementary material for: Unraveling Charging and Discharging Processes in Organic Radical‐Based Electrodes: A Hierarchical Molecular and Quantum Mechanical Approach
Source: ChemSusChem. 2026 Mar 15;19(6):e202502645. doi: 10.1002/cssc.202502645 (PMC12989221; doi:10.1002/cssc.202502645)
Supplement: Supplementary file 1 — Supplementary Material [file CSSC-19-e202502645-s001.pdf]

# Unravelling Charging and Discharging Processes in Organic Radical-based Electrodes – a Hierarchical Molecular and Quantum Mechanical Approach

Clara Zens[a]*, Georgina E. Shilito[a], Christian Friebe[b,c], and Stephan Kupfer[a]*

[a] Dr. C. Zens, Dr. G. E. Shillito, Dr. S. Kupfer

Institute of Physical Chemistry, Friedrich Schiller University Jena,

Helmholtzweg 4, 07743 Jena, Germany

E-Mail: clara.zens@uni-jena.de

E-mail: stephan.kupfer@uni-jena.de

[b] Dr. C. Friebe

Helmholtz Institute for Polymers in Energy Applications Jena (HIPOLE Jena), Lessingstraße 12-14, 07743 Jena, Germany

[c] Dr. C. Friebe

Helmholtz-Zentrum Berlin für Materialien und Energie, Hahn-Meitner-Platz 1, 14109 Berlin, Germany

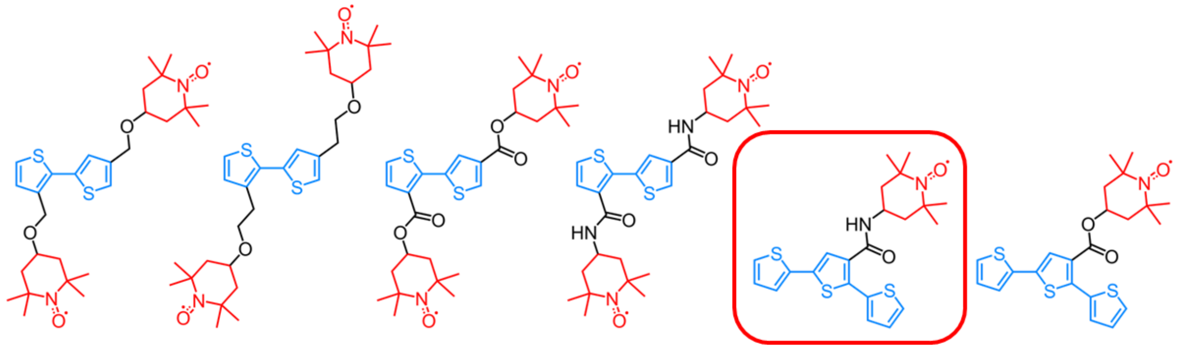

*Figure S1 Investigated monomers from our previous study.* [1] *TEMPO moieties are shown in red, the bithiophene and terthiophene backbone is shown in blue, the linker is shown in black. The monomer that served as basis for the herein investigated polymer is highlighted in red.*

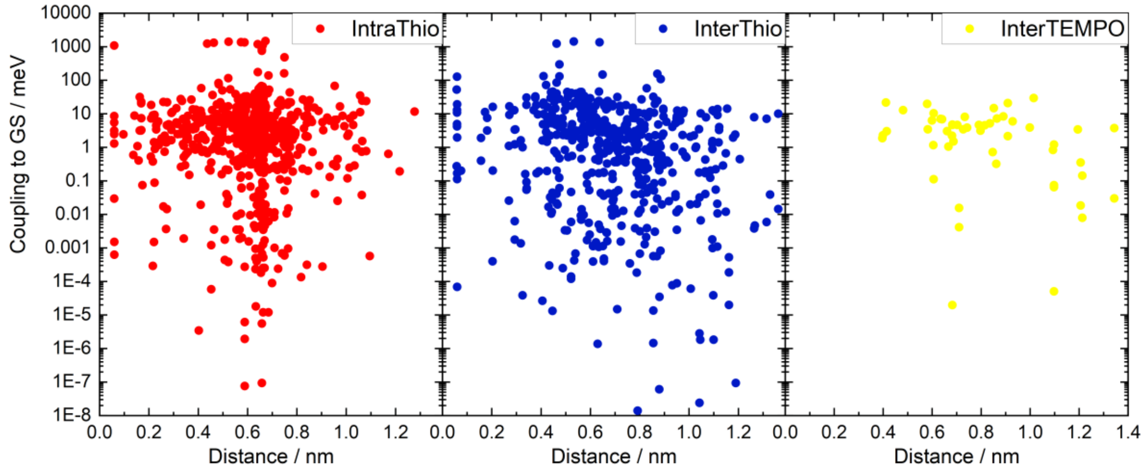


*Figure S2 Electronic coupling of snapshots at their respective distances. For better visibility a logarithmic scale was chosen for the couplings.*

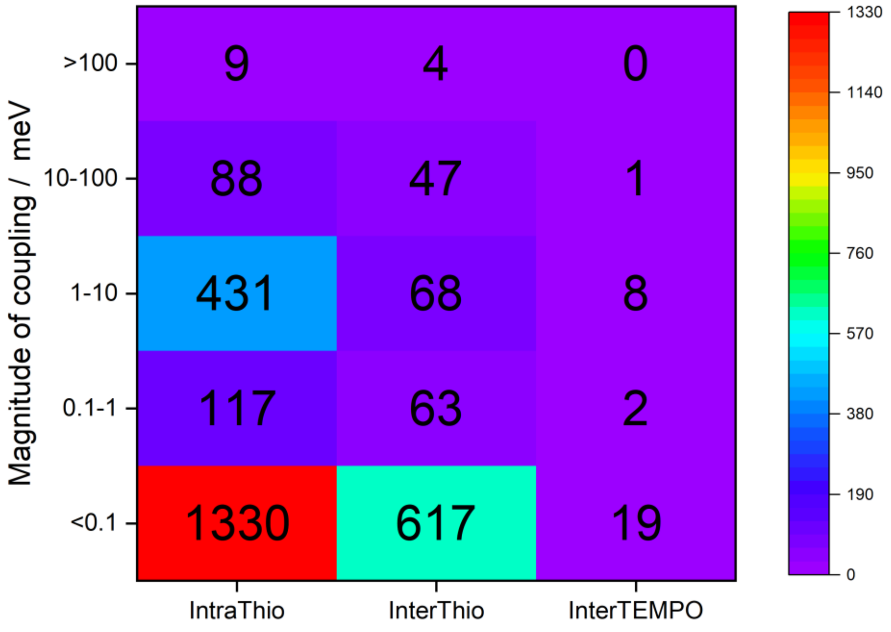


*Figure S3 Evaluated charge transfer (CT) processes in absolute numbers sectioned into intramolecular TEMPO-thiophene (IntraThio) CTs, intermolecular TEMPO-thiophene (InterThio) CTs and intermolecular TEMPO-TEMPO CTs (InterTEMPO).*

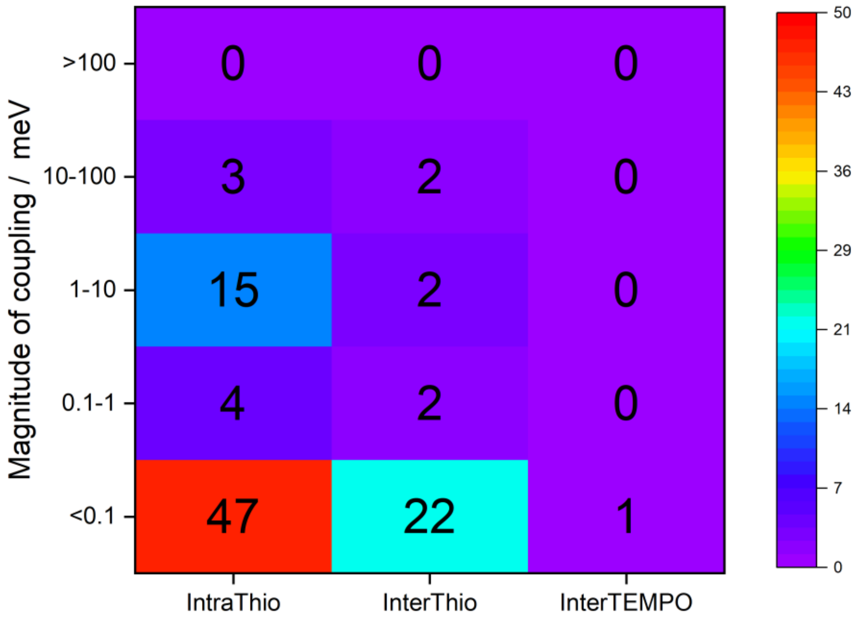


*Figure S4 Evaluated charge transfer (CT) processes in percent sectioned into intramolecular TEMPO-thiophene (IntraThio) CTs, intermolecular TEMPO-thiophene (InterThio) CTs and intermolecular TEMPO-TEMPO CTs (InterTEMPO).*

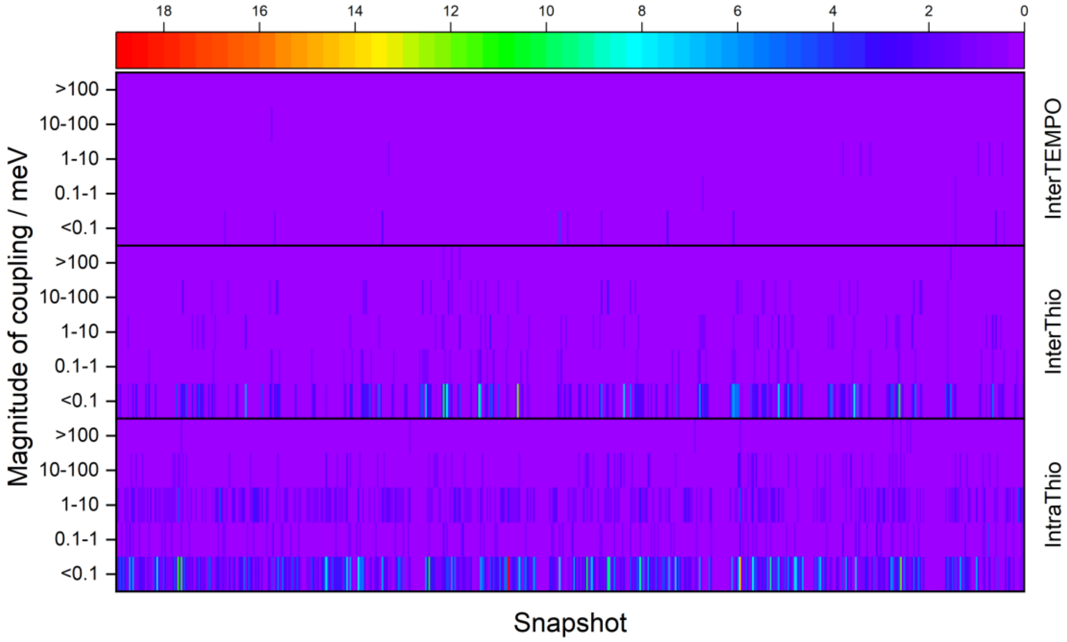


*Figure S5 Frequency and magnitude of couplings for intrastrand TEMPO-thiophene (IntraThio), interstrand TEMPO-thiophene (InterThio) and interstrand TEMPO-TEMPO (InterTEMPO) charge transfer processes for all evaluated snapshots. Colors of the heat map indicate the frequency of a specific type of transfer within one snapshot.*

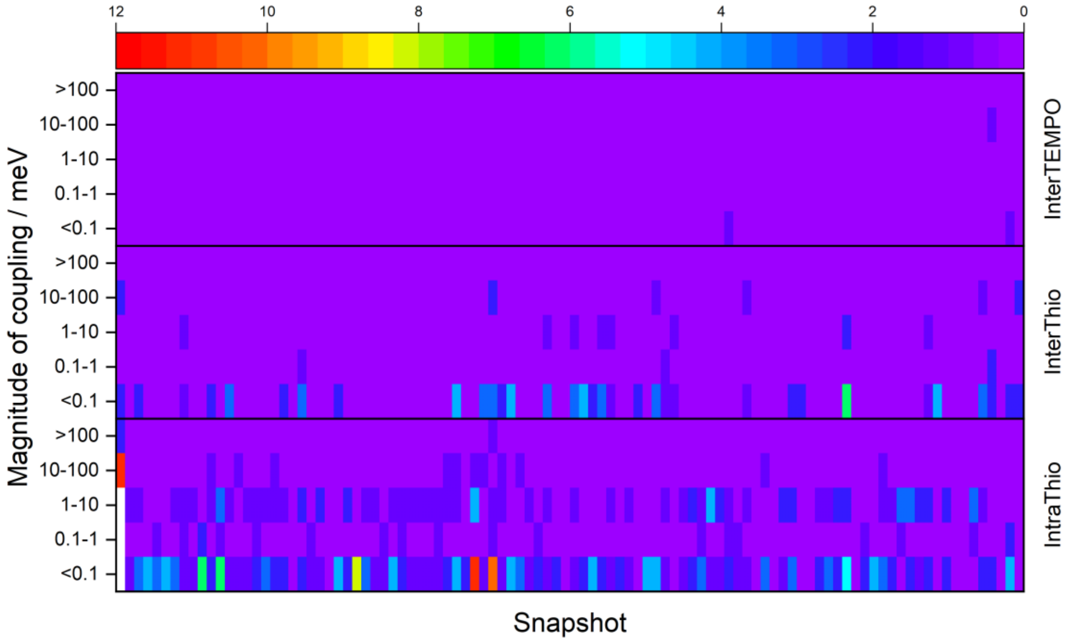


*Figure S6 Frequency and magnitude of couplings for intramolecular TEMPO-thiophene, intermolecular TEMPO-thiophene and intermolecular TEMPO-TEMPO charge transfer processes for different snapshots taken at t = 0 ns of the first production run. Colors of the heat map indicate the frequency of a specific type of transfer within one snapshot.*

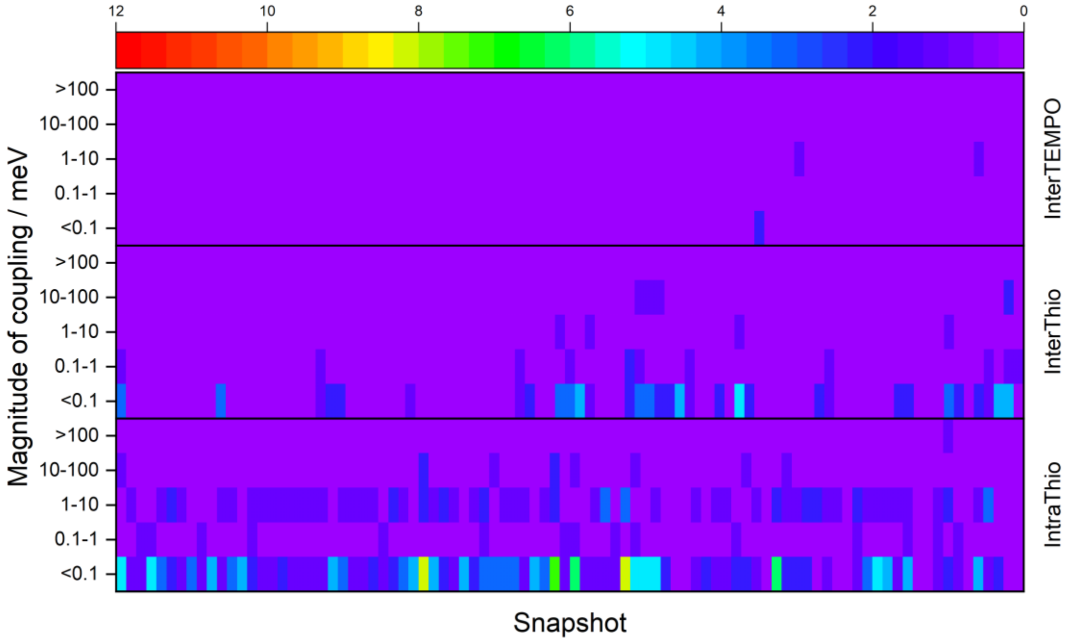


*Figure S7 Frequency and magnitude of couplings for intramolecular TEMPO-thiophene, intermolecular TEMPO-thiophene and intermolecular TEMPO-TEMPO charge transfer processes for different snapshots taken at t = 20 ns of the first production run. Colors of the heat map indicate the frequency of a specific type of transfer within one snapshot.*

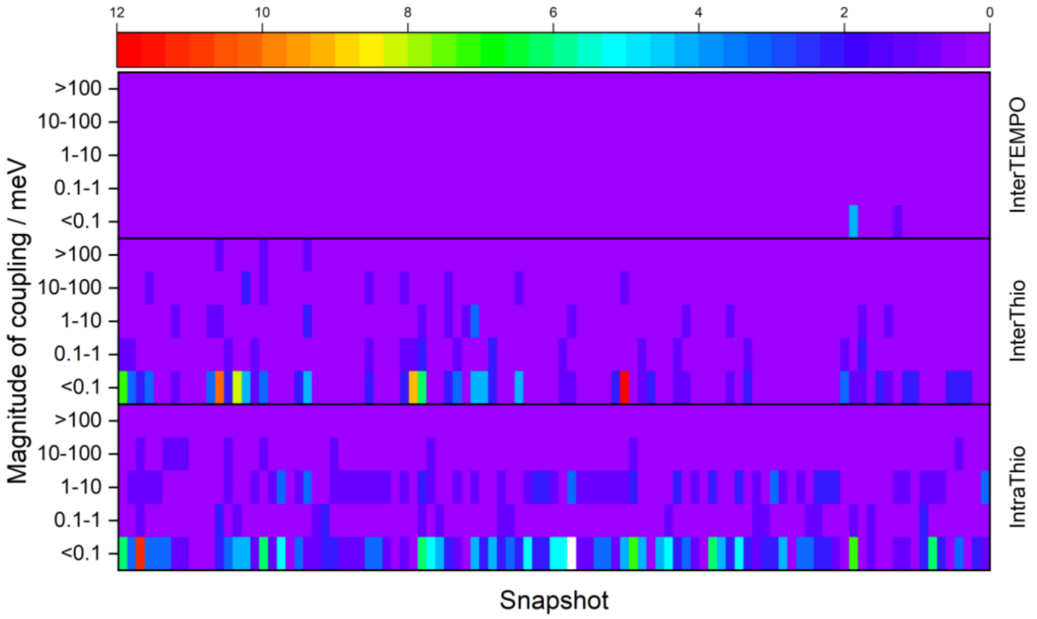


*Figure S8 Frequency and magnitude of couplings for intramolecular TEMPO-thiophene, intermolecular TEMPO-thiophene and intermolecular TEMPO-TEMPO charge transfer processes for different snapshots taken at t = 0 ns of the second production run. Colors of the heat map indicate the frequency of a specific type of transfer within one snapshot.*

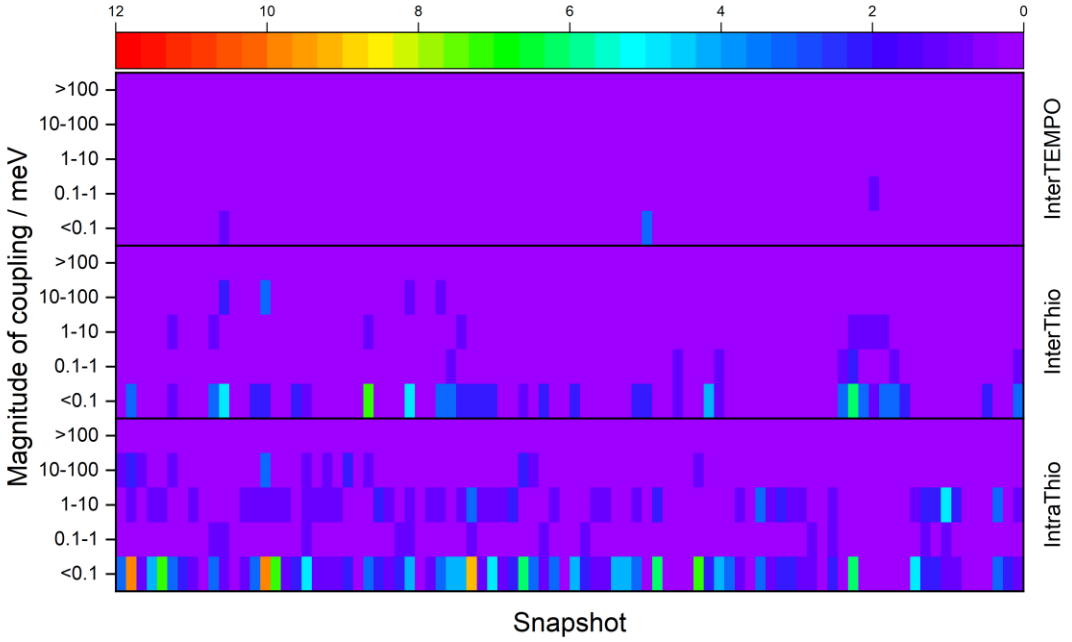


*Figure S9 Frequency and magnitude of couplings for intramolecular TEMPO-thiophene, intermolecular TEMPO-thiophene and intermolecular TEMPO-TEMPO charge transfer processes for different snapshots taken at t = 20 ns of the second production run. Colors of the heat map indicate the frequency of a specific type of transfer within one snapshot.*

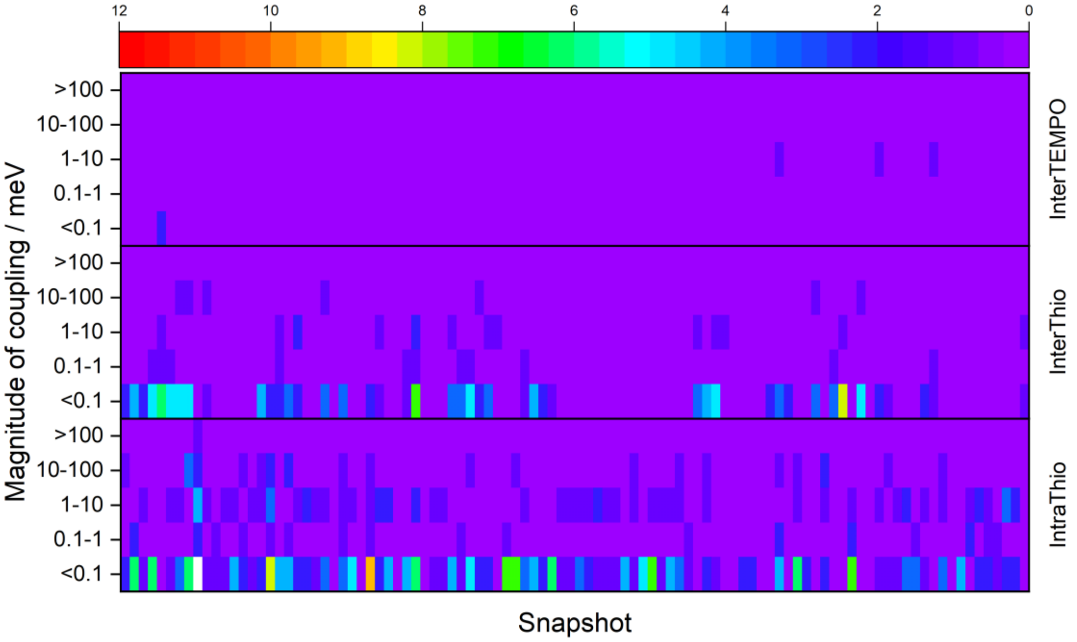


*Figure S10 Frequency and magnitude of couplings for intramolecular TEMPO-thiophene, intermolecular TEMPO-thiophene and intermolecular TEMPO-TEMPO charge transfer processes for different snapshots taken at t = 0 ns of the third production run. Colors of the heat map indicate the frequency of a specific type of transfer within one snapshot.*

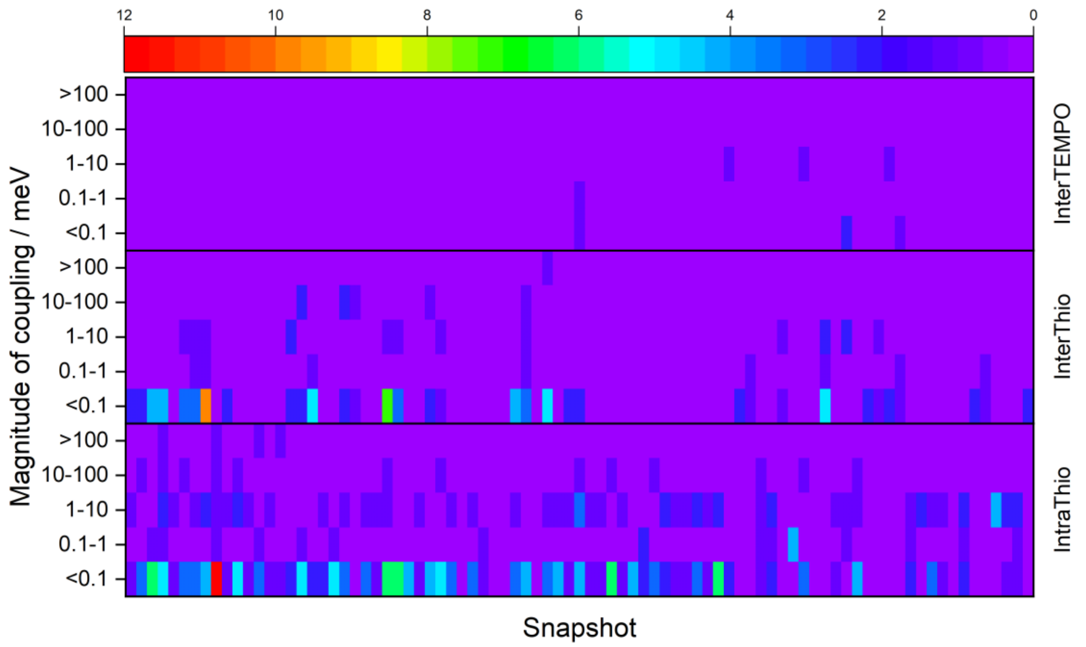


*Figure S11 Frequency and magnitude of couplings for intramolecular TEMPO-thiophene, intermolecular TEMPO-thiophene and intermolecular TEMPO-TEMPO charge transfer processes for different snapshots taken at t = 20 ns of the third production run. Colors of the heat map indicate the frequency of a specific type of transfer within one snapshot.*

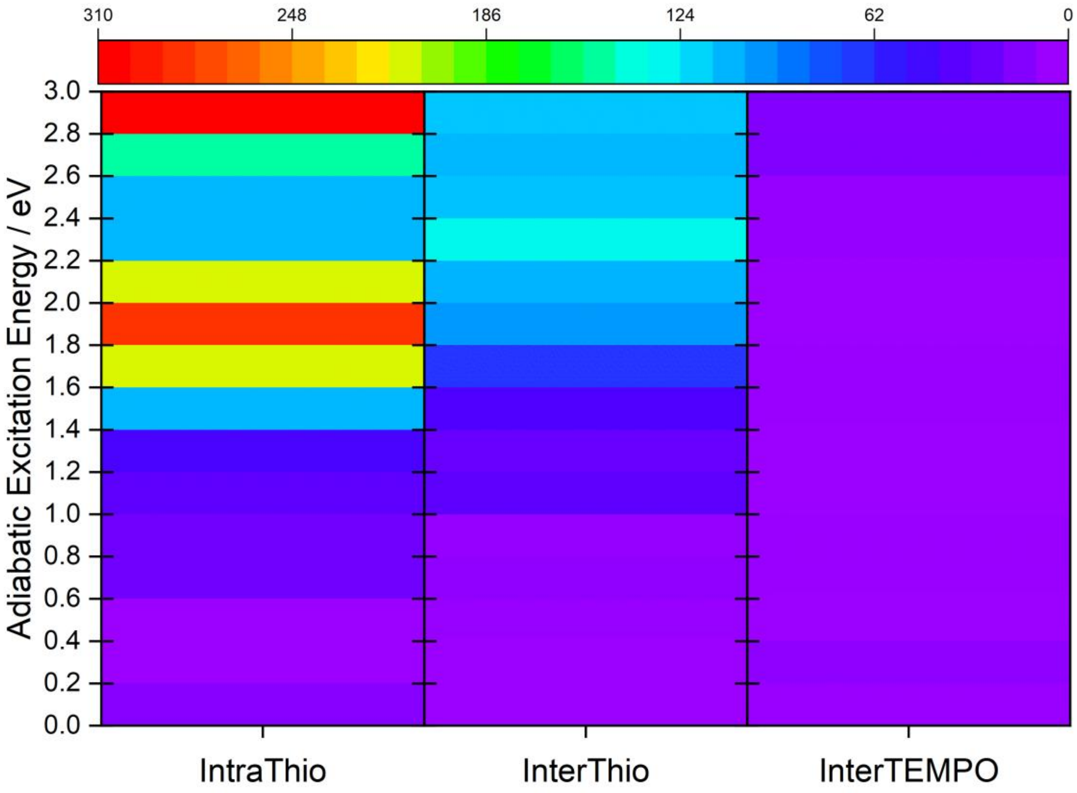


*Figure S 12 Occurrence of charge transfer (CT) processes attributed to the interstrand TEMPO-TEMPO (InterTEMPO) charge transfer (CT) type, the interstrand TEMPO-thiophene (InterThio) CT type and the intrastrand TEMPO-thiophene (IntraThio) type. Occurences of CTs are plotted relative to the respective adiabatic excitation energies of the CT. Occurences are indicated as absolute numbers represented as a heatmap in the color range from purple to red, where purple denotes no CTs and red 310 CTs.*

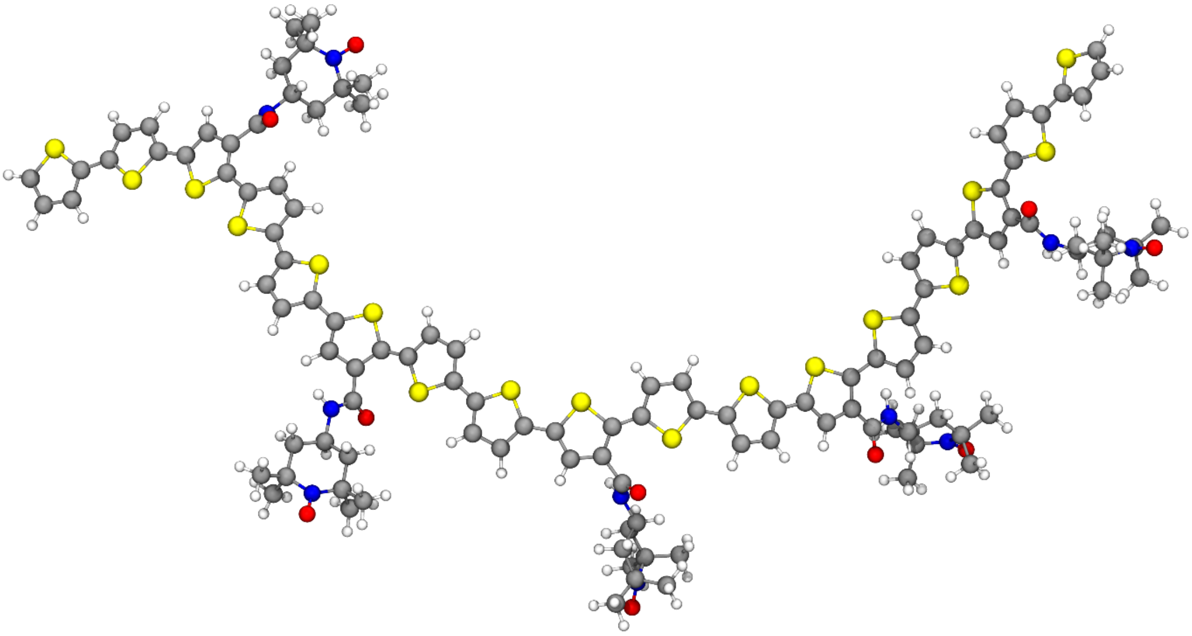

*Figure S13 Picture of one polymer strand of FNO.*

## References

[1] C. Zens, C. Friebe, U. S. Schubert, M. Richter, S. Kupfer, *ChemSusChem* **2023**, *16*, e202201679.
